# Semi‐Automatic Assessment of Crohn's Disease Activity by Combined Analysis of Bowel Lesions and Creeping Fat

**DOI:** 10.1002/mrm.70328

**Published:** 2026-03-07

**Authors:** Antoine Kneib, Astrée Lemore, Gabriela Hossu, Laurent Peyrin‐Biroulet, Valérie Laurent, Freddy Odille

**Affiliations:** ^1^ IADI (U1254), Université de Lorraine and Inserm Nancy France; ^2^ CIC‐IT 1433, Inserm, Université de Lorraine and CHRU Nancy Nancy France; ^3^ Service d'Hépato‐gastro‐entérologie CHRU Nancy Nancy France; ^4^ Nutrition‐Génétique et Exposition aux Risques Environnementaux (NGERE), Université de Lorraine Nancy France; ^5^ Département de Radiologie CHRU Nancy Nancy France

## Abstract

**Purpose:**

To develop and evaluate Crohn‐BOOST, an open‐source tool enabling semi‐automatic segmentation of intestinal lesions and creeping fat on magnetic resonance enterography (MR Enterography), and to assess whether quantitative metrics derived from these segmentations relate to radiological disease activity scored with simplified MaRIA (sMaRIA) and Nancy indices. A fully annotated MR Enterography dataset was also curated to support future artificial intelligence research.

**Methods:**

This retrospective single‐center study included 102 patients with Crohn's disease (134 analyzable small‐bowel lesions). Crohn‐BOOST was used to delineate bowel lesions and creeping fat. Extracted metrics included lesion volume, wall thickness, lesion length, ADC, arterial enhancement, and creeping fat volume. Radiologic activity was assessed with sMaRIA and Nancy scores. Spearman correlations (95% CI by bootstrap), multivariable linear regression, and fivefold cross‐validation were performed (*p* < 0.05).

**Results:**

Segmentation time was < 3 min per patient. Inter‐reader reproducibility in a two‐reader subset (*n* = 30) was high (Dice 0.85 for lesions; 0.87 for creeping fat). Lesion volume (sMaRIA *r* = 0.65; Nancy *r* = 0.61; *p* < 0.001) and ADC (sMaRIA *r* = −0.70) showed the strongest correlations with radiological activity. In multivariable models, these two metrics remained independent predictors, whereas creeping fat volume and enhancement metrics were not.

**Conclusion:**

Crohn‐BOOST enables fast, reproducible 3D quantification on routine MR Enterography and supports objective estimation of radiological disease activity. Lesion volume and ADC show strong associations with sMaRIA and Nancy scores, while creeping fat volume appears more related to chronic remodeling than acute inflammation.

## 1 Introduction

Crohn's disease (CD) is a chronic inflammatory condition of the gastrointestinal tract, characterized by segmental and discontinuous lesions [1]. A key feature of CD is the presence of mesenteric creeping fat, an expansion of mesenteric adipose tissue that envelops inflamed intestinal segments [2]. Beyond its structural role, creeping fat exerts immunomodulatory functions that may influence disease progression by modulating both inflammatory and fibrotic responses [3].

Recent studies have demonstrated that creeping fat is biologically active, with preadipocyte populations expressing pro‐inflammatory and profibrotic signatures and releasing mediators that promote smooth muscle proliferation and intestinal wall remodeling [4, 5]. Creeping fat has also been associated with an increased risk of complications in CD [4]. However, the relationship between creeping fat, intestinal lesion severity, and the distinction between active inflammation and fibrosis remains unclear [5].

Although ileocolonoscopy remains the reference standard for mucosal assessment in CD, it is limited to superficial and accessible segments of the bowel. Cross‐sectional imaging, particularly magnetic resonance imaging (MRI), is therefore complementary and offers the advantage of evaluating both mural and extramural disease, including small‐bowel segments beyond the reach of endoscopy [6]. In addition, MRI is a non‐invasive, radiation‐free modality suitable for longitudinal monitoring of disease activity.

Magnetic resonance enterography (MR Enterography) is an essential imaging modality for the evaluation and monitoring of CD [7]. Several MR Enterography‐based radiological activity indices have been proposed such as the MaRIA [8], simplified MaRIA (sMaRIA) [9], Nancy [10], and Clermont [11] scores. While these indices correlate well with endoscopic findings, they largely rely on qualitative or semi‐quantitative criteria and remain subject to inter‐ and intra‐observer variability, limiting their ability to reliably differentiate between inflammation and fibrosis [12].

Similarly, recent efforts to quantify creeping fat using radiological indices such as the mesenteric creeping fat index (MCFI) have demonstrated prognostic value in predicting treatment response and transmural healing [13, 14]. However, these approaches rely on visual grading applied to single 2D slices, introducing substantial inter‐observer variability and failing to capture the full three‐dimensional extent of mesenteric fat involvement. This highlights the need for volumetric, automated quantification methods that can provide objective and reproducible assessment of both intestinal lesions and associated creeping fat.

Quantitative and semi‐quantitative MRI (qMRI) approaches have the potential to provide robust and quantitative biomarkers of intestinal lesions [15]. When combined with morphological analysis such as bowel thickness, lesion length and creeping fat volume, these techniques could enhance the characterization of fibro‐inflammatory lesions [16]. To explore the relationship between creeping fat and intestinal lesions, accurate delineation of both regions is required from 3D imaging. While general‐purpose segmentation tools exist, they lack automated features specific to CD pathology. Several deep learning‐based approaches have been proposed for intestinal segmentation in CD [17, 18, 19]. However, these approaches either focus solely on lumen and wall delineation without addressing creeping fat, rely on CT imaging which involves radiation exposure, or employ custom severity grading systems not validated in clinical practice. To our knowledge, no existing method combines semi‐automated creeping fat segmentation with prediction of established radiological activity scores such as sMaRIA or Nancy, which are widely used for MR Enterography‐based CD assessment. Manual segmentation is time‐intensive and operator‐dependent, limiting reproducibility and hindering the creation of annotated datasets for deep learning.

To address these limitations, we developed Crohn‐BOOST (BOwel Open Source Tool), a semi‐automatic 3D Slicer plugin that provides precise segmentation of intestinal lesions and mesenteric creeping fat. From these segmentations, a broad set of morphological and functional imaging biomarkers were automatically extracted including lesion volume, creeping fat volume, bowel wall thickness, lesion length, ADC, and contrast enhancement. All measurements are standardized over three‐dimensional regions of interest.

Leveraging these quantitative features, we trained models to estimate disease activity as reflected by validated radiological activity scores, specifically the sMaRIA and Nancy indices. This approach enables objective and reproducible stratification of disease activity. Our objectives were threefold: (1) to provide an open‐source and reproducible tool for quantitative MRI analysis in CD; (2) to evaluate the ability of quantitative imaging features to approximate established radiological scores; (3) to generate a fully annotated MR Enterography dataset to support future research in artificial intelligence applied to inflammatory bowel disease (IBD).

## 2 Methods

### 2.1 Dataset Details and MR Protocol

In this retrospective, single‐center study, we included 102 consecutive patients with a confirmed diagnosis of Crohn's disease who underwent clinically indicated MR Enterography between April and September 2023, either for routine monitoring or evaluation of suspected disease flare. This retrospective study was approved by our institutional ethics committee (IRB), and informed consent was waived. Inclusion criteria required patients to have both an MR Enterography and a clinical assessment performed within a 3‐month interval. Clinical assessment included the Harvey‐Bradshaw Index (HBI), a validated clinical score assessing Crohn's disease activity based on general well‐being, abdominal pain, stool frequency, abdominal mass, and extraintestinal manifestations, and/or biomarkers such as C‐reactive protein (CRP) or fecal calprotectin. Exclusion criteria included age under 18 years, incomplete imaging protocol, isolated colonic or upper gastrointestinal disease, and lack of corresponding clinical data. The sample size was determined by the number of consecutive eligible patients meeting these criteria during the study period.

Patient demographics and clinical characteristics of the study cohort are summarized in Table 1. The mean age was 39.4±15.7 years, with 57 (55.9%) female patients. The mean body mass index (BMI) was $24.0\;\mathrm{kg}/m^{2}$ and 36 patients (35.3%) had a history of prior intestinal surgery.

**TABLE 1:** Demographic characteristics of the study cohort (*n* = 102).

Characteristics	Value
Mean age, years (±SD)	39.4±15.7
Female sex, *n* (%)	57(55.9%)
BMI, $\mathrm{kg}/m^{2}$ (mean ±SD)	24.0±4.5
Duration of disease, years (median [IQR])	12.0[5.25−19.5]
HBI score (mean ±SD)	2.9±3.3
CRP, mg/L	15.4±30.0(n=83)
Fecal calprotectin, μg/g	844±1101(n=42)
Prior intestinal surgery, *n* (%)	36(35.3%)
Number of current treatments (mean ±SD)	2.6±2.0
Biologic therapy, *n* (%)	72(70.6%)

*Note*: Values are expressed as mean ±standard deviation (SD) or median [interquartile range], as appropriate.

Abbreviations: BMI, body mass index; CRP, C‐reactive protein; HBI, Harvey‐Bradshaw Index.

Radiological activity scores, including sMaRIA and Nancy scores, were available for all patients.

The Nancy score [10] includes six binary criteria including diffusion hypersignal, early enhancement, mucosal‐submucosal dedifferentiation, bowel wall thickening, bowel wall edema and ulceration, each contributing equally to the total score. The sMaRIA score [9] aggregates four weighted features including bowel wall thickening, bowel wall edema, mesenteric fat infiltration and ulcerations. Both scores were assigned by the same radiologist (A.L.) during two separate reading sessions for each score, conducted at different time points and blinded to clinical and biological data, in order to minimize recall bias. Because lesion histopathology was not available in this retrospective cohort, validated MRI activity scores (sMaRIA and Nancy) were used as the reference standard.

MRI acquisition included a fat‐suppressed TRUFI sequence (SSFP) with a repetition time (TR) of 556ms, echo time (TE) of 2.14ms, flip angle (FA) of $60^{{}^{\circ}}$, slice thickness of 6 mm, slice spacing of 6mm, and an in‐plane resolution of $0.66\times 0.66\;{\mathrm{mm}}^{2}$. A T2‐weighted HASTE sequence (SSFSE) was also acquired with TR=1000ms, TE=81ms, $\mathrm{FA}=172^{{}^{\circ}}$, slice thickness = 5.5 mm, and in‐plane resolution of $1.04\times 1.04\;{\mathrm{mm}}^{2}$. Diffusion‐weighted imaging (DWI) was performed using a single‐shot echo planar imaging (SE‐EPI) sequence with $\mathrm{TR}=4400\;\mathrm{ms},\;\mathrm{TE}=46\;\mathrm{ms},\mathrm{FA}=90^{{}^{\circ}}$, slice thickness = 5 mm and in‐plane resolution of $1.27\times 1.27\;{\mathrm{mm}}^{2}$, with b‐values of $50,500\;\text{and}\;800\;s/{\mathrm{mm}}^{2}$ and signal averages (NEX) of 2, 5, and 12 respectively. Finally, a pre‐ and post‐contrast T1‐weighted VIBE sequence (3D spoiled gradient echo), with Dixon‐based water/fat separation, was acquired with TR = 6.51 ms, TE = 2.39 ms, FA = 11°, slice thickness = 3 mm and in‐plane resolution of $0.52\times 0.52\;{\mathrm{mm}}^{2}$. All acquisitions were performed on a 1.5 T MAGNETOM Avanto Fit system (Siemens Healthineers, Erlangen, Germany), following the standard clinical protocol of the center. All sequences were acquired during breath holding (exhale state), except the DWI sequence which was during free breathing.

All patients underwent standardized MR Enterography preparation according to the routine clinical protocol of the center. Oral contrast consisted of 1 L of mannitol administered over 45 min prior to imaging. An antispasmodic agent (0.5 mg intravenous glucagon) was administered at the time of contrast injection to reduce bowel peristalsis. Intravenous contrast‐enhanced imaging was performed using gadoteric acid (DOTAREM, Guerbet, France) at a dose of 0.2 mL/kg (0.5 mmol/mL), with image acquisitions obtained before injection and at 40 s, 80 s, and 3 min after injection. Lesions (defined as localized bowel segments showing imaging abnormalities on MR Enterography) were segmented on the last post‐contrast T1‐weighted VIBE images (3–4 min after injection), while mesenteric creeping fat was segmented as a single region per patient on the T2‐weighted HASTE sequence.

### 2.2 Segmentation Pipeline

#### 2.2.1 Slicer Plugin and Global Workflow

**FIGURE 1:**
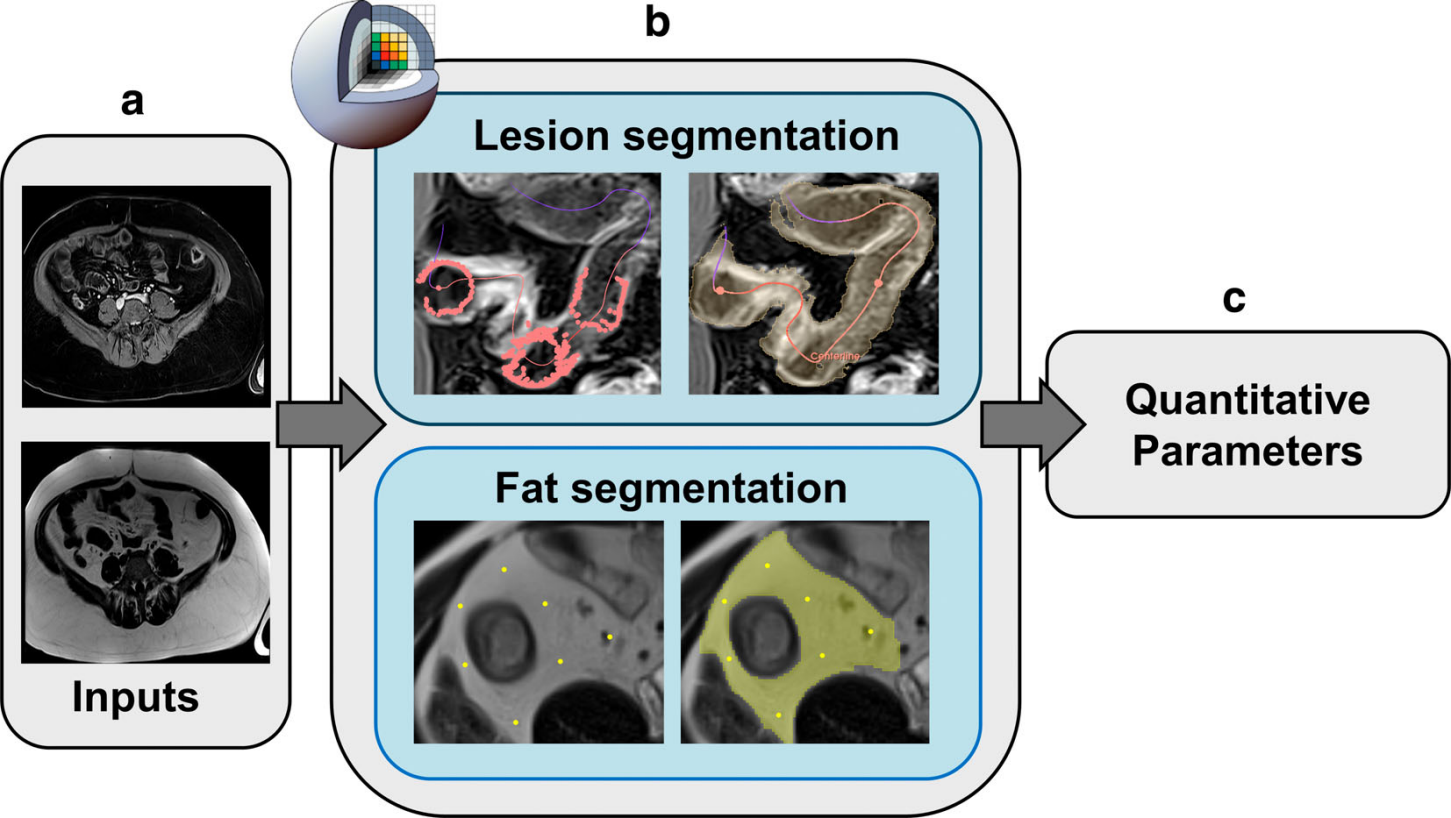
CrohnBOOST workflow for intestinal lesion and creeping fat segmentation and quantitative MRI biomarker extraction. (a) Input MRI sequences. (b) Semi‐automated segmentation in 3D Slicer: User‐drawn centerline along the intestinal lumen (red). Red dots represent candidate points automatically detected along radial rays, corresponding to the lumen‐bowel wall transition based on intensity gradient analysis. The semi‐transparent beige overlay corresponds to the final three‐dimensional segmentation of the intestinal wall derived from these candidate points. Seed points used to initiate creeping fat region growing are shown in yellow, with the resulting fat segmentation displayed as a yellow overlay. (c) Quantitative parameter extraction for clinical assessment.

Crohn‐BOOST was developed as a plugin, integrated into the open‐source 3D Slicer platform [20] https://www.slicer.org/ (version 5.6.2, 2024). The tool enables semi‐automatic, dual segmentation, targeting both the bowel wall lesion and the associated mesenteric creeping fat. The overall workflow is illustrated in Figure 1.

#### 2.2.2 Lesion Segmentation

To model the intestinal geometry, the user manually places N points along the intestinal lumen across different imaging planes. These points define a discrete polyline $\left\{p_{0},p_{1},\ldots ,p_{N}\right\}$, from which a cubic spline is interpolated to obtain a smooth centerline curve r(t), parameterized by t∈[0,1]. A regular arc‐length parameterization s(t) is then estimated as follows:

(1) $$s\left(t\right)=\int_{0}^{t}\left\|r^{\prime }\left(\tau \right)\right\|d\tau$$

This approach enables uniform sampling along the curve.

**FIGURE 2:**
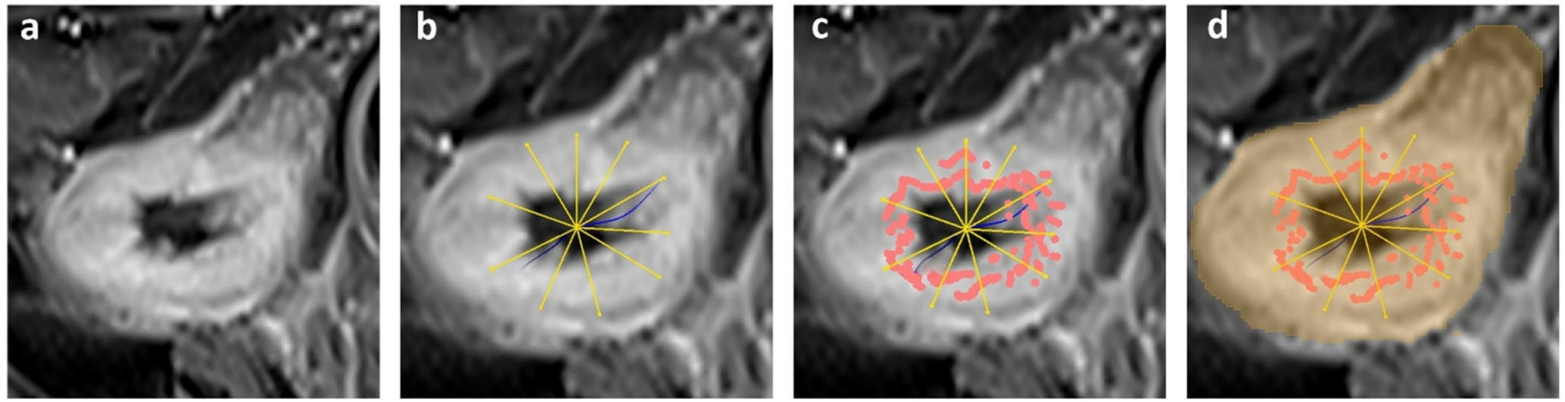
Stepwise illustration of the ray‐casting method for bowel wall segmentation. (a) Original MRI slice showing the affected bowel segment. (b) User‐defined centerline along the intestinal lumen (blue), with radial rays uniformly distributed around each sampled point. The centerline is a three‐dimensional structure; in this view, its local tangent is orientated partially out of the image plane, and the radial rays are generated within a plane orthogonal to this local direction. (c) Candidate points identified along each ray base on the maximum intensity gradient (red dots). (d) Final segmentation mask delineating the bowel wall (orange).

Thus, each position along the centerline curve r(t) is associated with an intrinsic coordinate s(t), corresponding to its arc‐length distance, allowing for consistent and reproducible analysis. Subsequently, each finely sampled position along this central curve serves as a spatial reference for local radial exploration using a ray‐casting technique, designed to precisely detect the boundary between the intestinal lumen and the bowel wall, similar to the RANSAC method [21]. Figure 2 illustrates these steps.

Each point $r\left(t_{i}\right)$ along the centerline is used as the origin of a radial sampling, uniformly distributed along 32 directions within the plane orthogonal to the local tangent, covering the full range of [0,2π]. Each ray is defined by:

(2) $$L_{\left(i,\theta \right)}\left(t\right)=r\left(t_{i}\right)+\mathrm{tR}\left(\theta \right){\vec{v}}_{⊥}$$

where R(θ) denotes a rotation of angle θ within the plane perpendicular to the tangential direction of the centerline, and ${\vec{v}}_{⊥}$ is a unit vector orthogonal to this tangent. The intensity along each ray is pre‐smoothed using a Gaussian filter (σ=0.5) to reduce noise influence. For each ray, the maximum intensity gradient ‖∇I(x)‖ is automatically detected within a user‐defined search window (adjustable via a slider), and is considered as the lumen‐wall transition point. This extraction of candidate points delineates the boundary between the bowel wall and the intestinal lumen.

This detection is formalized by the following function:

(3) $$\text{mask}\left(L_{\left(i,\theta \right)}\left(t\right)\right)=\left\{\begin{array}{l} 1,\text{if}\;\left\|\nabla I\left(L_{\left(i,\theta \right)}\left(t\right)\right)\right\|\geq G_{\text{threshold}}\\ 0,\text{otherwise} \end{array}\right.$$

where I(x) represents the intensity of the voxel at position $x\in {\mathbb{R}}^{3}$, ∇I(x) is the spatial intensity gradient at that point, and $G_{\text{threshold}}$ is a threshold value used to detect abrupt variations characterizing tissue‐lumen interfaces.

After identifying the candidate points marking the lumen‐wall transition through intensity gradient analysis along the radial rays, a constrained adaptive expansion segmentation procedure is applied. Starting from this point cloud, a region‐growing algorithm is initiated within an anisotropic three‐dimensional window (8×8×2voxels), calibrated according to the spatial resolution of the MRI acquisitions.

The inclusion of a voxel v is based on a decision function that combines both spatial and intensity criteria:

(4) $$f\left(v\right)=\left\{\begin{array}{l} 1\;\text{if}\;d_{C}\left(v\right)\leq r_{\text{lumen}}\\ 1\;\text{if}\;d_{P}\left(v\right)\leq r_{\exp}\wedge I\left(v\right)\in \left[\mu -k\sigma ,\mu +k\sigma \right]\\ 0\;\text{otherwise} \end{array}\right.$$

where $d_{C}\left(v\right)$ and $d_{P}\left(v\right)$ represent the minimum Euclidean distances from voxel v to the centerline and the detected wall points, respectively. The parameter $r_{\text{lumen}}$ corresponds to the estimated radius of the intestinal lumen, while $r_{\exp}$ defines the maximum expansion radius estimated around the wall points (adjustable via the user interface). The intensity I(v) is compared to a statistical range centered on the mean intensity μ of the detected wall points, with a fixed tolerance of k=2, corresponding to the interval [μ−2σ,μ+2σ].

To ensure complete inclusion of the intestinal lumen, voxels located within the contour defined by the bowel wall are automatically incorporated using a 3D morphological filling algorithm. A post‐processing step is then applied, including an anisotropic binary closing (with a structuring element of 2×2×3) and the removal of disconnected components representing less than 5% of the main volume, to ensure the topological consistency of the final segmentation.

#### 2.2.3 Fat Segmentation

**FIGURE 3:**
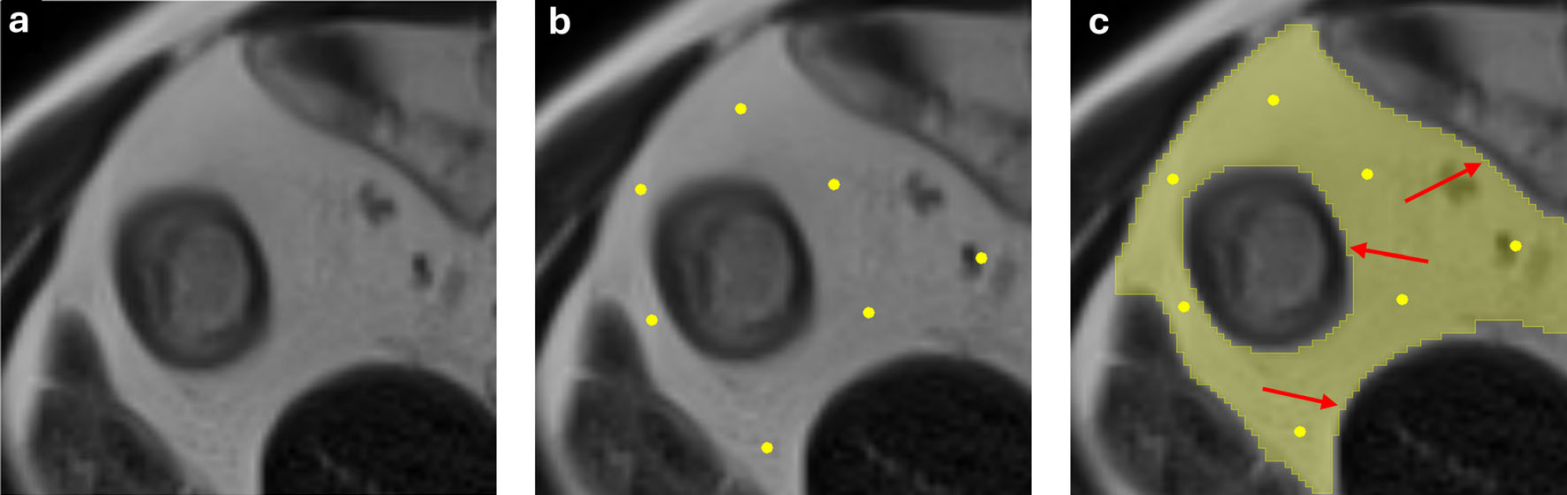
Stepwise process for creeping fat segmentation. (a) Original MRI slice without annotation. (b) User‐placed seed points (yellow) initiating the segmentation of creeping fat. (c) Final segmentation mask of creeping fat (yellow), with red arrows highlighting the peripheral boundaries where fat interfaces with adjacent structures.

The segmentation of perilesional adipose tissue or creeping fat is directly integrated into our 3D Slicer plugin, allowing for rapid and guided annotation around the previously identified intestinal lesions. This step relies on a semi‐automatic interaction, where the user places a set of points $Q=\left\{q_{0},q_{1},\ldots ,q_{M}\right\}$, with each $q_{i}\in {\mathbb{R}}^{3}$, used as seeds to initiate and guide a controlled region‐growing process, as illustrated in Figure 3.

The process accounts for the anisotropic characteristics of the MRI volumes by decomposing the expansion into two successive phases: first, propagation within the axial plane (XY), followed by restricted extension along the longitudinal direction (Z). At each iteration k, the segmented region $R_{k}$ is updated according to:

(5) $$R_{k+1}=R_{k}\cup \left\{v\in N\left(R_{k}\right):I\left(v\right)\in \left[\mu -2\sigma ,\mu +2\sigma \right]\wedge v\notin \right.\left.\;M_{\text{lesion}}\right\}$$

where $N\left(R_{k}\right)$ denotes the immediate neighborhood of $R_{k},I\left(v\right)$ is the intensity of voxel v, μ and σ represent the mean and standard deviation of the intensities of the seed points, and $M_{\text{lesion}}$ is the intestinal lesion mask to be excluded. The user can iteratively refine the segmentation by adding new seed points as needed.

The peripheral boundary of the creeping fat was identified using anatomical, morphological, and intensity‐based criteria. Creeping fat was defined as hypertrophied mesenteric adipose tissue that expands asymmetrically around inflamed bowel segments, displacing adjacent structures and creating discernible interfaces with surrounding tissues. The segmentation naturally terminated when neighboring voxels exhibited an intensity profile incompatible with adipose tissue, particularly at the junctions with other abdominal organs or mesenteric structures. Its presence was often associated with characteristic fat‐filled spaces, sometimes accompanied by displacement of nearby bowel loops, which are commonly used radiological features to support creeping fat identification on MR images. Because creeping fat does not have a sharp anatomical boundary on MRI, strict voxel‐level separation from adjacent adipose tissue cannot be guaranteed.

**FIGURE 4:**
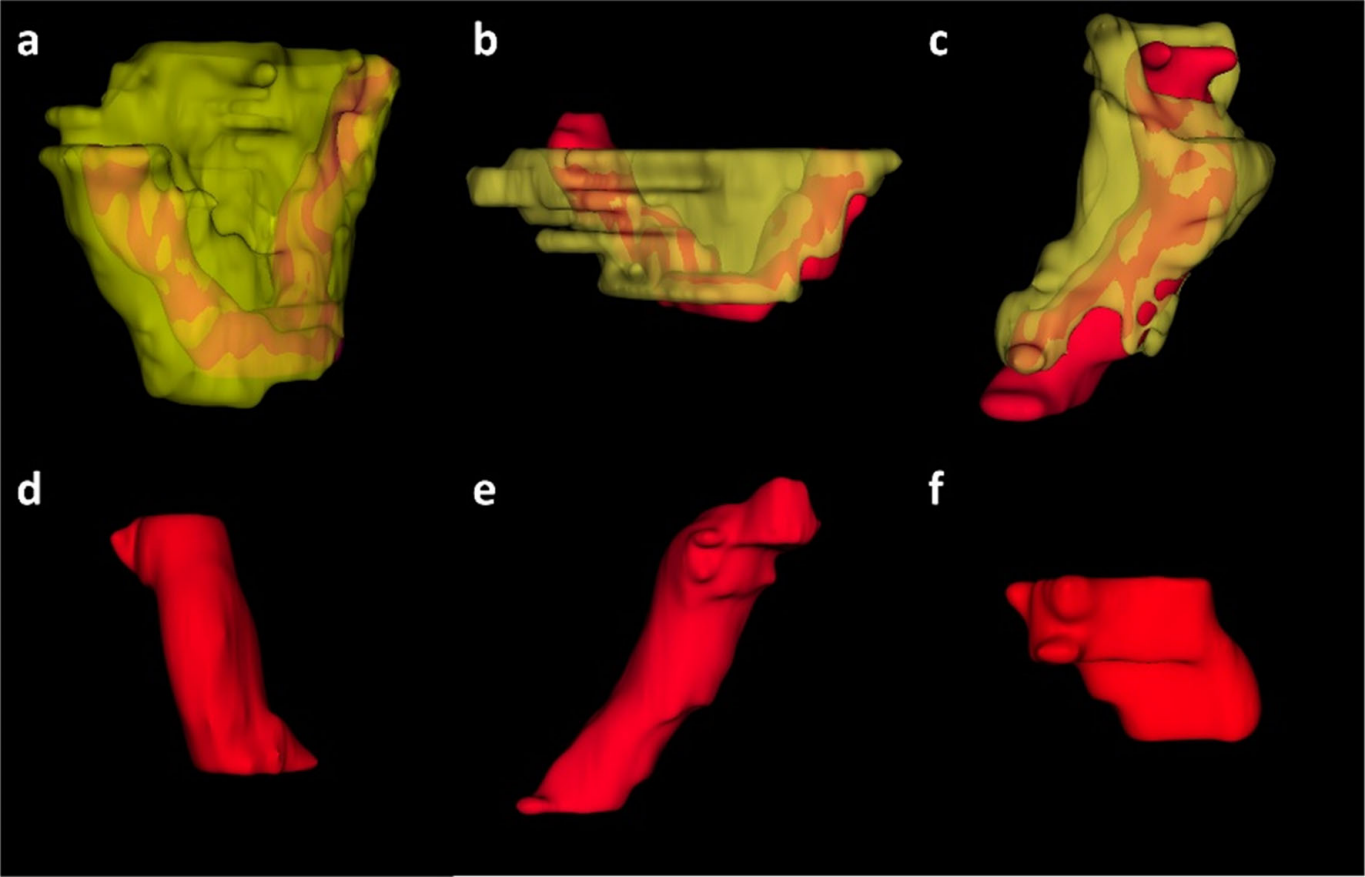
Examples of segmented bowel lesions with and without creeping fat. (a–c) Bowel lesions with surrounding creeping fat. (d–f) Bowel lesions without creeping fat, showing absence of adjacent fat segmentation.

Connected‐component post processing removed isolated regions. Figure 4 illustrates typical 3D renderings of segmented bowel lesions with and without surrounding creeping fat.

#### 2.2.4 Lumen‐Wall Separation

The separation between the intestinal lumen and the bowel wall was performed using a Graphcut segmentation algorithm [17, 18], applied directly within the native lesion volume. The centerline obtained during the lesion segmentation step was used to guide this separation by providing a spatial prior on lumen location. This step allows for the precise distinction between luminal regions and wall regions, facilitating the extraction of wall‐specific quantitative parameters.

The problem is formulated as the minimization of an energy function E(L), defined for each voxel p∈P as follows:

(6) $$E\left(L\right)=\sum_{p\in P}D\left(L_{p}\right)+\lambda \sum_{\left\{p,q\right\}\in N}V\left(L_{p},L_{q}\right)$$

where $L_{p}\in \left\{\text{lumen},\text{wall}\right\}$ represents the label assigned to voxel p, N denotes the set of neighboring voxel pairs, and λ controls the trade‐off between data fidelity and spatial regularity. The data attachment term is defined as $D\left(L_{p}\right)=-\log\left(\mathrm{Pr}\left(L_{p}|I_{p},d_{p}\right)+\varepsilon \right)$, where $I_{p}$ denotes the intensity of voxel p, and $d_{p}$ its distance to the centerline, which acts as a spatial prior favoring lumen labels close to the centerline and wall labels at larger distances.

The conditional probabilities $V\left(L_{p},L_{q}\right)=\left\{\begin{array}{l} \exp\left(-\frac{\left|I_{p}-I_{q}\right|}{A}\right)\\ 0 \end{array}\right.$ correspond to the regularization term. It promotes smooth segmentation and prevents abrupt label changes, particularly at region boundaries. This binary segmentation provides an accurate delineation of the bowel wall regions, from which quantitative measurements (including thickness, intensity, enhancement, ADC etc.) are extracted. When necessary, the segmentation could be manually corrected by the radiologist. The spatial regularization ensures smooth boundaries and compensates for small irregularities caused by noise or motion artifacts. An illustrative example of the lumen‐wall separation obtained with this approach is shown in Figure S1.

### 2.3 Reproducibility of the Segmentation

A junior radiologist (A.L.) with 3 years of experience in abdominal MRI segmented the whole database. Additionally, a subset of 30 datasets were also segmented by a PhD student (A.K.) with 1 year of experience in abdominal MRI. The second reader was informed of the lesion location identified by the radiologist but was blinded to all clinical and biological data, as well as to the segmentations performed by the first reader. This was done to evaluate the reproducibility of the segmentation tool itself, independently of the reproducibility of radiologist identifying the lesions. The agreement between segmentations from the two readers was assessed by Dice similarity coefficients (DSC) scores for the lesions and creeping fat. The duration of the segmentation process for the second reader was also recorded.

### 2.4 Quantitative Parameters Extraction

All DWI volumes $\left(b=50,500\;\text{and}\;800\;s/{\mathrm{mm}}^{2}\right)$ were first reformatted into the space of the delayed post‐contrast T1‐weighted VIBE (DIXON) sequence by interpolation, using information from DICOM coordinates.

To further ensure precise anatomical alignment before ADC computation, a dedicated image registration and preprocessing pipeline was applied. First, the diffusion‐weighted image acquired with $b=50\;s/{\mathrm{mm}}^{2}$ was non‐rigidly registered to the delayed post‐contrast T1‐weighted VIBE (DIXON) sequence, which served as the anatomical reference. Registration was performed using the pTVreg library, applying the localized cross‐correlation metric, via MATLAB [19].

The resulting deformation field was then propagated to the higher b‐value images $\left(b=500\;\text{and}\;b=800\;s/{\mathrm{mm}}^{2}\right)$, before ADC map computation by linear regression of the following equations:

(7) $$\mathrm{ADC}=-\frac{\ln\left(S_{b}\right)-\ln\left(S_{0}\right)}{b}$$

where $S_{b}$ corresponds to the signal intensity at a given diffusion weighting (b‐value), and $S_{0}$ is the signal intensity without diffusion weighting [22]. Additionally, all phases of the DCE T1 VIBE sequence were registered to the delayed phase using the same registration framework.

The relative enhancement $\left({\mathrm{RE}}_{\text{phase}}\right)$ was calculated from dynamic T1 VIBE sequences acquired before $\left(S_{\mathrm{pre}}\right)$ and after gadolinium injection during each phase (early arterial, venous and delayed phases) $\left(S_{\text{phase}}\right)$, using the following formula:

(8) $${\mathrm{RE}}_{\text{phase}}=\frac{S_{\text{phase}}-S_{\mathrm{pre}}}{S_{\mathrm{pre}}}\times 100$$

The intestinal wall thickness was measured by calculating, for each cross‐sectional slice, the mean distance between the internal boundary (lumen‐wall interface) and the external boundary (wall‐mesenteric fat interface) according to the following formula:

$$\text{Thickness}\left(s\right)=R_{\mathrm{int}}\left(s\right)-R_{\mathrm{ext}}\left(s\right)$$

The final bowel wall thickness value was calculated as the mean thickness across the entire affected intestinal segment using the centerline.

The total volume of each segmented region (bowel lesion and creeping fat) was computed from the corresponding three‐dimensional binary masks according to

$$V=N_{\text{voxels}}\times ∆x\times ∆y\times ∆z$$

where $N_{\text{voxels}}$ denotes the number of voxels belonging to the segmentation mask, and ∆x, ∆y, and ∆z correspond to the voxel dimensions extracted from image metadata (in‐plane pixel spacing and inter‐slice spacing). Volumes are reported in ${\mathrm{cm}}^{3}$.

### 2.5 Statistical Analysis

Statistical analyses were performed using Python, specifically with the Pandas and SciPy libraries. Normally distributed continuous variables are expressed as means, standard deviations, minimum, maximum, non‐normally distributed data as medians and interquartile range and categorical variables as frequencies (percentages). Correlations between quantitative MRI parameters and MRI scores were assessed using the Spearman method with bootstrapping (10 000 iterations) to obtain confidence intervals. We tested the null hypothesis of no correlation. The normality of quantitative variables (ADC, RE, thickness, etc.) was assessed using the Shapiro–Wilk test. A *p* value <0.05 was considered statistically significant.

We assessed how well quantitative MRI markers (including lesion volume, wall thickness, ADC, enhancement, and creeping fat volume) could approximate or explain variance in sMaRIA and Nancy scores. Multiple linear regression models were constructed to assess individual and combined contributions of these variables.

### 2.6 Segmentation Tool Availability

The segmentation tool described in this study was implemented as a dedicated module for the 3D Slicer platform (version ≥ 5.6.2). Developed in Python and leveraging the VTK, VMTK, NumPy, SimpleITK and SciPy libraries, this plugin allows for full integration within the Slicer environment. The source code and user documentation are made available under an open‐source MIT license at the following address: https://github.com/AntoineKneib/CrohnBOOST (commit SHA‐1: 88133a5).

## 3 Results

### 3.1 Usability and Reproducibility of Crohn‐BOOST for Segmentation

All segmentations were performed using the Crohn‐BOOST plugin within 3D Slicer. Segmentation time per case ranged from 1 min 16 s to 4 min 31 s, depending on the presence of creeping fat. Cases with creeping fat required more time (1 min 50s—4 min 31 s) than cases without (1 min 16 s—2 min 32 s), reflecting the added complexity of perilesional fat annotation.

Reproducibility was evaluated on a subset of 30 patients, as described in the Methods. The mean Dice coefficient for bowel lesion segmentation was 0.85 overall; 0.81±0.15 in creeping fat‐positive lesions and 0.89±0.05 in creeping fat‐negative cases. For creeping fat masks, the mean Dice was 0.87±0.04.

### 3.2 Cohort Characteristics

A total of 102 patients with CD were included in the study, contributing 134 analyzable small‐bowel lesions.

At the time of MRI, the mean HBI score was 2.9±3.3. Inflammatory markers were variably available, with a mean CRP of 15.4±30.0mg/L (*n* = 83) and a mean fecal calprotectin of 844±1101μg/g (*n* = 42). Radiological disease activity, assessed per‐lesion, showed a mean sMaRIA score of 5.8±3.3 and a mean Nancy score of 6.8±4.3. Creeping fat was present in 56 of 134 lesions (41.8%) with a mean creeping fat volume of $162.4\pm 114.8\;{\mathrm{cm}}^{3}$ when present.

Complete demographic and clinical characteristics are summarized in Table 1. Detailed descriptive statistics of the annotated dataset, including MRI activity scores, ADC values, and volumes of interest for bowel lesions and creeping fat, are reported in Table 2.

**TABLE 2:** Imaging characteristics of the annotated MR enterography dataset at the patient level.

Variable	Value
sMaRIA score	5.8±3.3
Nancy score	6.8±4.3
ADC $\left(\times 10^{-3}{\mathrm{mm}}^{2}/s\right)$	1.50±0.36
VOI size—bowel lesions $\left({\mathrm{cm}}^{3}\right)$	49±41
VOI size—creeping fat $\left({\mathrm{cm}}^{3}\right)$	162±115

*Note*: Values are expressed as mean ±standard deviation. For patients with multiple bowel lesions, lesions volumes and MRI activity scores were summed to derive patient‐level metrics, whereas ADC values were averaged across lesions.

Abbreviations: ADC, apparent diffusion coefficient; VOI, volume of interest.

### 3.3 Association of MRI Biomarkers With Radiological Disease Activity

**FIGURE 5:**
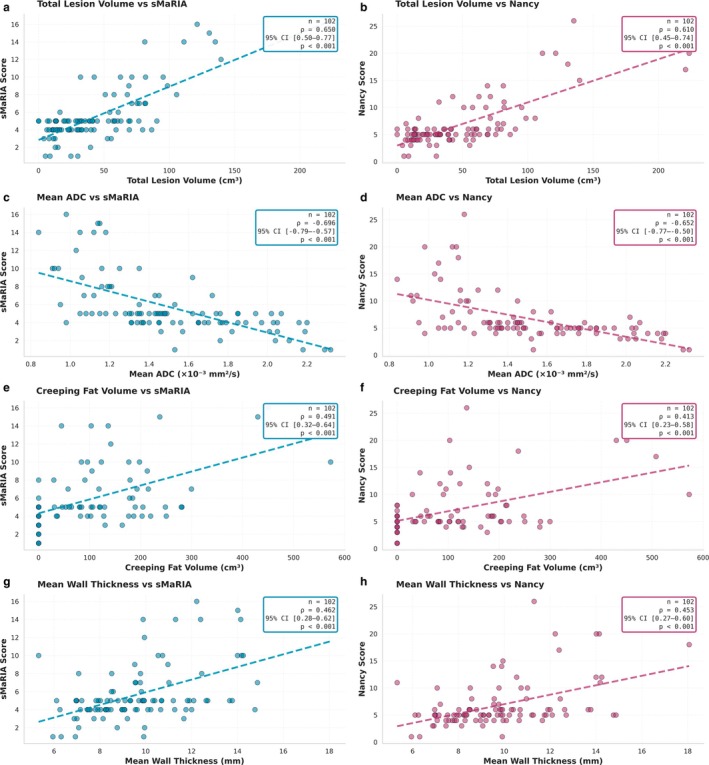
Correlations between quantitative MRI parameters and disease activity scores. (a, b) Total lesion volume versus sMaRIA and Nancy scores. (c, d) Mean ADC versus sMaRIA and Nancy scores. (e, f) Creeping fat volume versus sMaRIA and Nancy scores. (g, h) Mean wall thickness versus sMaRIA and Nancy scores. Scatter plots show patient‐level correlations (*n* = 102) with fitted regression lines (dashed). All correlations were statistically significant (*p* < 0.001). Statistics boxes display Spearman correlations coefficients (ρ) with 95% confidence intervals (bootstrap, 10.000 iterations) and *p* value.

All quantitative MRI parameters showed statistically significant, but variably strong, correlations with both the sMaRIA and Nancy scores, as shown in Figure 5. Lesion volume showed strong correlation with sMaRIA (r=0.65,95%CI[0.505:0.765],p<0.001) and Nancy (r=0.61,95%CI[0.455;0.739],p<0.001). Mean ADC was negatively correlated with both sMaRIA (r=−0.70,95%CI[−0.792;−0.566],p<0.001) and Nancy (r=−0.65,95%CI[−0.767;−0.504],p<0.001).

Moderate correlations were observed between creeping fat volume and both sMaRIA (r=0.49,95%CI[0.323;0.636], p<0.001). Lesion length (sMaRIA: r=0.50,95%CI[0.324;0.658],p<0.001) and bowel wall thickness (sMaRIA:r=0.46,95%CI[0.280;0.620],p<0.001) were also positively associated with sMaRIA.

Relative enhancement parameters showed weaker correlations. Arterial enhancement correlated with sMaRIA (r=0.28,95%CI[0.100;0.453],p=0.004), and delayed enhancement correlated with Nancy (r=0.28,95%CI[0.084;0.452], *p* = 0.005).

### 3.4 Correlation of MRI Activity Scores With Clinical and Biological Markers

Radiological scores showed statistically significant but variable correlations with clinical and biological markers of inflammation. As shown in Table 3, both sMaRIA and Nancy scores were moderately correlated with the HBI (r=0.36andr=0.37,respectively;bothp<0.001). Fecal calprotectin also showed correlations with sMaRIA (r=0.33,p=0.03) and Nancy (r=0.36,p=0.02) scores.

**TABLE 3:** Correlation between MRI‐derived parameters and clinical and biological markers of disease activity.

MRI parameter	Harvey‐Bradshaw Index (HBI)	CRP	Fecal calprotectin
Radiological activity scores			
sMaRIA score	0.36(p<0.001)	0.27(p<0.01)	0.33(p<0.05)
Nancy score	0.37(p<0.001)	NS	0.36(p<0.05)
Quantitative imaging parameters			
Mean ADC $\left(\times 10^{-3}{\mathrm{mm}}^{2}/s\right)$	−0.29(p<0.01)	−0.33(p<0.01)	NS
$\text{Lesion volume}\;\left({\mathrm{cm}}^{3}\right)$	NS	NS	0.36(p<0.05)
Creeping fat volume $\left({\mathrm{cm}}^{3}\right)$	NS	NS	NS

*Note*: Values are Spearman correlation coefficients (r) with corresponding *p* values.

Abbreviations: ADC, apparent diffusion coefficient; CRP, C‐reactive protein; HBI, Harvey‐Bradshaw Index; NS, not significant.

sMaRIA correlated weakly with CRP (r=0.27,p=0.01), whereas no significant correlation was observed between CRP and the Nancy score. Mean ADC values showed significant negative correlations with both HBI (r=−0.29,p=0.003) and CRP (r=−0.30,p=0.006), consistent with restricted diffusion in active inflammation.

### 3.5 Multivariable Prediction Models for Radiological Activity

We developed linear regression models using quantitative MRI biomarkers to predict radiological activity scores in 102 patients. For both sMaRIA and Nancy scores, models included total lesion volume, mean ADC, creeping fat volume, total lesion length, mean bowel wall thickness, and arterial/delayed enhancement parameters as candidate predictors.

Although the final model included three predictors (lesion volume, mean ADC and creeping fat volume), only lesion volume and mean ADC reached statistical significance, while creeping fat volume did not (p>0.05). The full multivariable regression results, including regression coefficients (β), 95% confidence intervals, and *p* values for all predictors included in the final models, are reported in Table 4. For sMaRIA, lesion volume (β=0.047, 95% CI [0.023–0.071], *p* < 0.001) and mean ADC (β=−2.59, 95% CI [−3.87; −1.30], *p* < 0.001) were significantly associated. For Nancy score, lesion volume (β=0.068,95%CI[0.033−0.102],p<0.001) and mean ADC (β=−2.42,95%CI[−4.30;−0.55],p=0.012) remained significant predictors. Creeping fat volume, lesion length, wall thickness, and enhancement parameters showed no independent association (all *p* > 0.05).

**TABLE 4:** Multivariable linear regression models for prediction of radiological activity scores.

Variable	β (sMaRIA)	95% CI	*p*	β (Nancy)	95% CI	*p*
Lesion volume $\left({\mathrm{cm}}^{3}\right)$	0.047	[0.023;0.071]	<0.001	0.068	[0.033;0.102]	<0.001
Mean ADC $\left(\times 10^{-3}{\mathrm{mm}}^{2}/s\right)$	−2.59	[−3.87;−1.30]	<0.001	−2.42	[−4.30;−0.55]	<0.05
Creeping fat volume $\left({\mathrm{cm}}^{3}\right)$	−0.0001	[−0.005;0.004]	0.980	−0.005	[−0.011;0.002]	0.153

*Note*: Multivariable linear regression coefficients (β), 95% confidence intervals (CI), and *p* values for quantitative MRI biomarkers included in the final patient‐level models predicting the sMaRIA and Nancy scores. Models were adjusted for lesion volume, mean apparent diffusion coefficient (ADC), and creeping fat volume. β coefficients represent the change in radiological activity score per unit increase in each predictor. ADC is expressed in $\times 10^{-3}\;{\mathrm{mm}}^{2}/s$.

**FIGURE 6:**
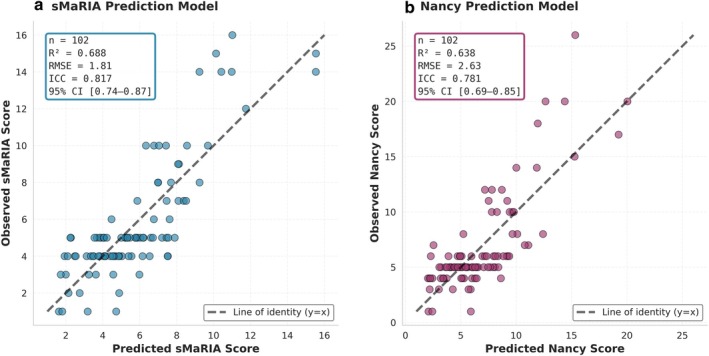
Multivariate model performance: Predicted vs. observed activity scores. (A) sMaRIA prediction model. (B) Nancy prediction model. Scatter plots show agreement between predicted and observed scores at patient level (*n* = 102) using multivariate linear regression with three quantitative MRI parameters. Dashed line represents perfect prediction (line of identity *y* = *x*). Both models demonstrated good performance with statistics boxes display model performance metrics.

Both models demonstrated good predictive performance (Figure 6). The sMaRIA model explained 69% of the variance ($R^{2}=0.69,\text{adjusted}\;R^{2}=0.66;\text{RMSE}=1.81$), with excellent agreement between predicted and observed scores (ICC=0.82,95%CI[0.75−0.87]). Fivefold cross‐validation confirmed model stability (mean RMSE=1.99±0.19). The Nancy model explained 64% of the variance ($R^{2}=0.64$, adjusted $R^{2}=0.61;\text{RMSE}=2.63$), with good agreement (ICC=0.79,95%CI[0.71−0.85]) and cross‐validation RMSE of 2.93±0.61. As shown in Figure 6, predicted and observed values were symmetrically distributed around the identity line, indicating no systematic bias.

## 4 Discussion

In this study, we present Crohn‐BOOST, an open‐source, semi‐automated MR Enterography tool that performs simultaneous 3D segmentation of bowel‐wall lesions and adjacent creeping fat. From these segmentations, the pipeline derives reproducible quantitative biomarkers, including lesion volume and length, bowel wall thickness, ADC, relative enhancement, and creeping fat volume. We then use these key features to predict radiologic activity defined by the sMaRIA and Nancy indices, with the goal of automating score computation from objective, quantitative features derived from the same imaging data. This approach does not aim to predict independent clinical outcomes, but rather to reproduce validated radiological indices in a standardized manner. To our knowledge, this is the first work to couple volumetric creeping fat quantification with MR Enterography‐based activity estimation using validated indices, supporting a more objective and reproducible assessment of CD activity.

In our cohort of 102 patients with 134 small‐bowel lesions, Crohn‐BOOST enabled dual segmentation of lesions and adjacent creeping fat in under 3 min per case, with DSC of 0.84 for lesions and 0.87 for creeping fat. Quantitative MR Enterography measurements including lesion volume and ADC showed strong correlations with radiological activity (sMaRIA:ρ=+0.65and−0.70respectively).

In multivariable models, only lesion volume and mean ADC remained independent predictors of both sMaRIA and Nancy ($R^{2}=0.69\;\text{and}\;0.64,\text{respectively}$). Volume integrates wall thickness and lesion length, explaining why these components did not remain independently significant. This finding aligns with recent work showing that volumetric assessment correlates with endoscopic severity and treatment response [23]. The inverse association between ADC and activity aligns with prospective studies and meta‐analyses [24]. Correlations with biological markers were moderate.

In contrast, relative‐enhancement parameters including arterial/delayed enhancement were not significantly correlated with radiologic activity and did not retain independent value in multivariable models. Prior work has highlighted that contrast‐based measures are phase‐ and normalization‐dependent [25]. These factors may limit their reproducibility across protocols and centers; by contrast, diffusion‐weighted imaging is timing‐independent and has shown good diagnostic performance for active inflammation [26, 27, 28].

A specific limitation of our enhancement analysis is the use of a single wall‐averaged relative‐enhancement value. Crohn's lesions often display layered mural enhancement, which a wall‐average cannot capture. To our knowledge, automated depth‐resolved quantification on MR Enterography has not yet been reported in CD. Future work should develop and evaluate metrics based on wall depth derived from 3D wall models to determine whether this information adds incremental value [29].

Although creeping fat volume showed moderate correlations with disease activity in univariate analysis, it was not independently associated with activity scores after adjustment. These findings suggest that creeping fat reflects chronic perilesional remodeling (fibrosis and muscular hypertrophy) rather than acute inflammation. These findings are consistent with prior studies associating creeping fat with fibrostenotic changes and long‐standing disease [30]. While not a standalone marker of inflammation, creeping fat remains a valuable feature to contextualize disease severity. Because creeping fat expands asymmetrically along the mesenteric axis and not as a concentric peri‐bowel layer, fixed‐distance definitions were not used. In this study, creeping fat was identified using established MR Enterography features, as surgical or histopathological confirmation was not systematically available.

Inflammatory and fibrotic components frequently coexist within the same CD lesion, making their strict separation challenging using imaging alone. While the present study does not allow a definitive distinction between inflammation and fibrosis in the absence of histopathological correlation, histopathology‐correlated MR Enterography studies have shown that ADC reflects both inflammatory edema and fibrotic tissue composition [31]. The present work therefore provides a quantitative, multiparametric framework that could support future investigations aiming to better characterize fibro‐inflammatory disease.

While segmental indices such as the Clermont score [11] integrate ADC together with wall thickness, edema, and ulcers—and show performance comparable to MaRIA for endoscopic lesions—our aim was different: to provide a patient‐level estimate of overall activity by aggregating diffusion and morphological measurements across all lesions, using the sMaRIA and Nancy frameworks. Adding another segmental score (such as Clermont) would have been redundant and potentially collinear with features already modeled (notably ADC and wall thickness); a single patient‐level summary is better aligned with risk stratification and longitudinal monitoring [32, 33].

Recent deep‐learning‐based segmentation methods show promise, but performance depends on large, annotated datasets that remain scarce in routine MR Enterography [34]. Crohn‐BOOST addresses this practical gap by delivering consistent dual segmentation of the bowel wall and creeping fat and by exporting standardized masks and measurements suitable for dataset curation and benchmarking. Implemented as a 3D Slicer extension, the pipeline provides a reproducible framework for semi‐automatic quantitative analysis (volume, length, thickness, ADC, enhancement, creeping fat volume) that is usable at the point of care while also serving as a generator of high‐quality annotations for training and validating automated algorithms [20].

Several limitations should be acknowledged. First, the retrospective, single‐center design and moderate sample size may introduce selection bias and limit generalizability. Second, all examinations were acquired on a single 1.5 T system with a uniform protocol; quantitative readouts such as relative enhancement can vary across scanners and field strengths, underscoring the need for cross‐platform verification [35]. Third, although the tool reduces manual effort, it remains semi‐automatic and requires user interaction (seed placement, parameter tuning).

Importantly, our approach differs from prior semi‐automated MR Enterography methods in both scope and clinical framing. Earlier tools typically targeted a single measurement for example, semi‐automatic bowel‐wall delineation to derive thickness or relied on CPR workflows with CNN assistance to segment lumen/wall and facilitate measurements; these systems still required manual refinement and were largely segment‐centric (often pediatric), without modeling creeping fat or providing a patient‐level activity estimate anchored to validated indices [17, 36, 37]. By contrast, Crohn‐BOOST performs simultaneous 3D segmentation of the bowel wall and adjacent creeping fat, derives a panel of quantitative features (volume, length, thickness, ADC, enhancement, creeping fat volume), and reports a single patient‐level estimate of disease burden consistent with sMaRIA/Nancy, which is better suited to risk stratification and longitudinal monitoring [38].

Finally, the pipeline is backbone‐agnostic: it accepts automatic pre‐segmentations and can integrate future DL models. To confirm metric stability and foster broader adoption, we will pursue multi‐scanner and multi‐center validation, ideally leveraging public MR Enterography datasets [39], a need underscored by the cross‐platform variability of quantitative readouts [35].

## 5 Conclusion

Crohn‐BOOST provides an open‐source, semi‐automatic framework for 3D quantification of bowel lesions and creeping fat on MR Enterography. In 102 patients (134 lesions), the tool enabled fast and reproducible dual segmentation and extraction of quantitative biomarkers. Lesion volume and ADC were the strongest predictors of radiological disease activity $\left(R^{2}=0.69\;\text{for sMaRIA}\;\text{and}\;0.64\;\text{for Nancy}\right).$ Creeping fat volume did not contribute independently in multivariable models. These findings support a patient‐level estimate of disease burden suited to longitudinal monitoring. Reproducibility was encouraging in a small two‐reader subset (n=30). Further validation in larger, external cohorts is warranted. Crohn‐BOOST also enabled the curation of an annotated MR Enterography dataset that may facilitate future methodological developments.

## Funding

This work was funded by the Agence Nationale de la Recherche (ANR) under grant numbers ANR‐23‐RHUS‐0016, ANR‐23‐IAHU‐0012, and by Métropole du Grand Nancy. It was conducted on a platform co‐funded by the French government through the Contrat de Plan Etat‐Région (CPER 2015‐2020 IT2MP) and by the European Regional Development Fund (ERDF 2014‐2020). The platform is affiliated with the France Life Imaging (ANR‐11‐INBS‐0006).

## Disclosure

The authors have nothing to report.

## Conflicts of Interest

Laurent Peyrin‐Biroulet reports the following conflicts of interest related to his professional activities (not necessarily related to the present manuscript): He has received personal fees from AbbVie, Allergan, Alma, Amgen, Applied Molecular Transport, Arena Pharmaceuticals, Biogen, Bristol Myers Squibb (BMS), Boehringer Ingelheim, Celgene, Celltrion, Enterome, Enthera, Ferring Pharmaceuticals, Fresenius Kabi, Galapagos, Genentech, Gilead, Hikma, InDex Pharmaceuticals, Inotrem, Janssen, Lilly, MSD, Mylan, Nestlé, Norgine, OSE Immunotherapeutics, Oppilan Pharma, Pfizer, Pharmacosmos, Roche, Samsung Bioepis, Sandoz, Sterna, Sublimity Therapeutics, Takeda, Theravance, Tillots, and Vifor Pharma.

## Supporting information


**Figure S1:** Bowel wall delineation using the Graphcut algorithm. (a) Native post‐contrast T1‐weighted MR Enterography image of an inflamed bowel segment. (b) Fully automatic lumen‐wall separation obtained with the Graphcut algorithm, without manual correction, showing the segmented intestinal lumen (yellow) and bowel wall (green), guided by the centerline (red). This delineation enables extraction of wall‐specific quantitative parameters such as thickness, signal intensity enhancement, and ADC.

## Data Availability

The source code and user documentation for Crohn‐BOOST are available under an open‐source MIT license at: https://github.com/AntoineKneib/CrohnBOOST (commit SHA‐1: 88133a5). No imaging data or patient‐level datasets can be shared due to institutional and regulatory restrictions.
