# Supplementary material for: Semi‐Automatic Assessment of Crohn's Disease Activity by Combined Analysis of Bowel Lesions and Creeping Fat
Source: Magn Reson Med. 2026 Mar 7;96(1):302–14. doi: 10.1002/mrm.70328 (PMC13156446; doi:10.1002/mrm.70328)
Supplement: Supplementary file 1 — Figure S1: Bowel wall delineation using the Graphcut algorithm. (a) Native post‐contrast T1‐weighted MR Enterography image of an inflamed bowel segment. (b) Fully automatic lumen‐wall separation obtained with the Graphcut algorithm, without manual correction, showing the segmented intestinal lumen (yellow) and bowel wall (green), guided by the centerline (red). This delineation enables extraction of wall‐specific quantitative parameters such as thickness, signal intensity enhancement, and ADC. [file MRM-96-302-s001.docx]

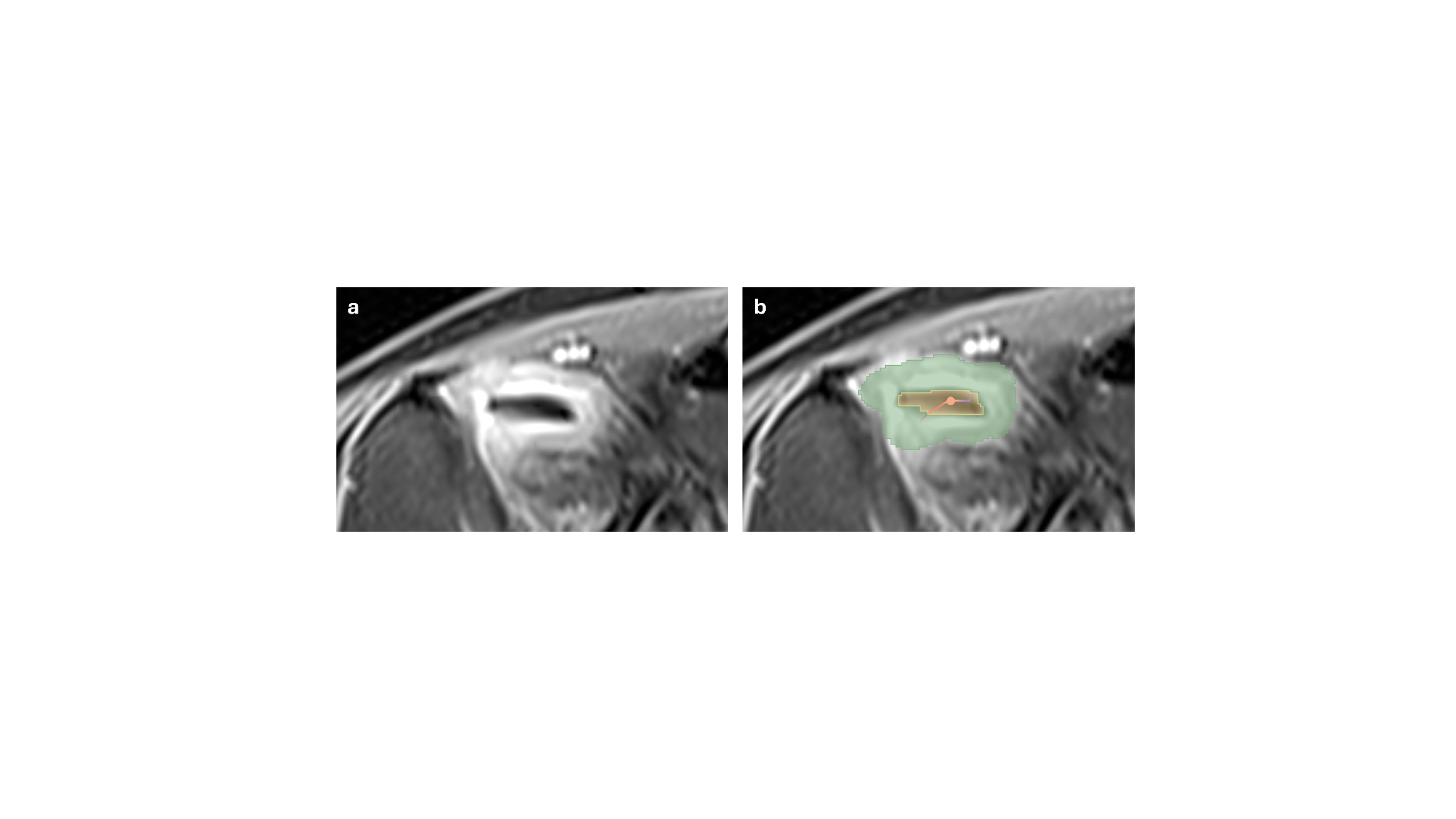


**Figure S1.** Bowel wall delineation using the Graphcut algorithm. a) Native post-contrast T1-weighted MR Enterography image of an inflamed bowel segment. b) Fully automatic lumen-wall separation obtained with the Graphcut algorithm, without manual correction, showing the segmented intestinal lumen (yellow) and bowel wall (green), guided by the centerline (red). This delineation enables extraction of wall-specific quantitative parameters such as thickness, signal intensity enhancement, and ADC.
